# Crosstalk between Irisin Levels, Liver Fibrogenesis and Liver Damage in Non-Obese, Non-Diabetic Individuals with Non-Alcoholic Fatty Liver Disease

**DOI:** 10.3390/jcm11030635

**Published:** 2022-01-27

**Authors:** Angelo Armandi, Chiara Rosso, Aurora Nicolosi, Gian Paolo Caviglia, Maria Lorena Abate, Antonella Olivero, Daphne D’Amato, Marta Vernero, Melania Gaggini, Giorgio Maria Saracco, Davide Giuseppe Ribaldone, Diana Julie Leeming, Amalia Gastaldelli, Elisabetta Bugianesi

**Affiliations:** 1 Department of Medical Sciences, University of Turin, 10126 Turin, Italy; aurora.nicolosi@unito.it (A.N.); gianpaolo.caviglia@unito.it (G.P.C.); marialorena.abate@unito.it (M.L.A.); antonella.olivero@unito.it (A.O.); daphne.damato@unito.it (D.D.); marta.vernero@gmail.com (M.V.); giorgiomaria.saracco@unito.it (G.M.S.); davrib_1998@yahoo.com (D.G.R.); elisabetta.bugianesi@unito.it (E.B.); 2 Metabolic Liver Research Program, University Medical Center, Department of Internal Medicine I, Johannes Gutenberg University, 55131 Mainz, Germany; 3 Division of Gastroenterology, Città della Salute e della Scienza University-Hospital, 10100 Turin, Italy; 4 Cardiometabolic Risk Unit, Institute of Clinical Physiology, CNR, 56121 Pisa, Italy; mgaggini@ifc.cnr.it (M.G.); amalia@ifc.cnr.it (A.G.); 5 Nordic Bioscience, 2730 Herlev, Denmark; djl@nordicbio.com

**Keywords:** irisin, insulin resistance, non-invasive biomarkers, liver fibrosis, liver fibrogenesis, PRO-C3, PRO-C6, NAFLD

## Abstract

Background: Insulin resistance plays a relevant role in the onset of non-alcoholic fatty liver disease (NAFLD) and its progression to non-alcoholic steatohepatitis (NASH) and fibrosis. Irisin is an exercise-induced myokine involved in the regulation of energy homeostasis and glucose metabolism. Additionally, pre-clinical models have shown a potential role of irisin in the pathogenesis of NAFLD. The aim of this study is to explore the association between irisin, histological features and biomarkers of liver fibrogenesis in non-diabetic, non-obese, biopsy-proven NAFLD individuals. Methods: Forty-one patients with histological evidence of NAFLD were included. Circulating irisin and direct markers of fibrogenesis N-terminal type III collagen propeptide (PRO-C3) and type VI collagen cleavage product (PRO-C6) were measured by ELISA. Results: Median age of the cohort was 45 years (41–51) and 80.4% were male. Significant fibrosis (stage ≥ 2) was present in 36.6% of cases. Circulating irisin, PRO-C3 and PRO-C6 levels were significantly higher in subjects with fibrosis stage ≥ 2 when compared to those with fibrosis stage < 2 (5.96 ng/mL (95% CI = 4.42–9.19) vs. 2.42 ng/mL (95% CI = 1.73–5.95), *p* = 0.033; 9.5 ng/mL (95% CI = 7.7–13.6) vs. 6.2 ng/mL (95% CI = 4.9–8.9), *p* = 0.016; 6.6 ng/mL (95% CI = 5.6–7.9) vs. 5.1 ng/mL (95% CI = 4.2–5.4), *p* = 0.013, respectively). Irisin levels were similarly distributed between the features of NASH. Circulating irisin positively correlated with both PRO-C3 and PRO-C6 levels (r = 0.47, *p* = 0.008 and r = 0.46, *p* = 0.002). Conclusions: Increased circulating irisin levels may identify a more aggressive phenotype of liver disease with increased fibrogenesis and more severe liver damage.

## 1. Introduction

Non-Alcoholic Fatty Liver Disease (NAFLD) is currently the most common chronic liver disease, tightly associated with type 2 diabetes mellitus (T2DM) and the metabolic syndrome (MetS). Liver necro-inflammatory changes superimposed to fat accumulation (Non-Alcoholic Steatohepatitis, NASH) leads to scar tissue deposition in the parenchyma (fibrosis) and to the progression towards end-stage liver disease and its complications. Liver fibrosis is the most relevant prognostic factor and its early recognition is a mainstay of management in NAFLD patients [1]. 

Insulin resistance (IR) in the adipose tissue, liver and skeletal muscle (SM) is a well-recognized pathophysiological mechanism underlying both the onset and progression of NAFLD [2]. The study of the metabolic cross-talks between insulin-sensitive tissues can unveil potential targets for biomarkers or therapy. Irisin is a recently discovered cytokine mainly synthesized in the SM in response to physical exercise and it is involved in energy metabolism, favoring thermogenesis and browning of adipose tissue [3]. Irisin is also produced in the adipose tissue, where it regulates lipid metabolism and glucose uptake [4] and may be involved in the regulation of pancreatic beta-cell activity [5]. 

In animal studies, irisin reduced gluconeogenesis and stimulated glycogen synthesis [6], reduced cholesterol content from hepatocytes of both lean and obese mice [7] and modulated oxidative stress [8]. These findings are remarkable, as oxidative stress is the main source of lipid-driven hepatocyte damage, leading to chronic inflammation and ultimately to fibrogenesis. However, studies conducted on NAFLD individuals have yielded conflicting results. Zhang et al. found that serum irisin levels are inversely associated with intrahepatic fat content detected by magnetic resonance spectroscopy (MRS) [9], also confirmed by Metwally et al., in patients with liver biopsy [10]. On the contrary, another study found a positive association between irisin levels and steatosis, NASH and liver fibrosis [11]. Finally, a recent meta-analysis reported that irisin levels are increased in mild NAFLD with respect to moderate–severe NAFLD, but specifically in the Asian population [12]. 

In this study, we assessed the association between circulating irisin levels and metabolic parameters of IR, markers of fibrogenesis and histological fibrosis in a well-characterized cohort of biopsy-proven NAFLD individuals in the absence of major metabolic confounders (obesity and T2DM). 

## 2. Materials and Methods

### 2.1. Study Population

This is a retrospective, cross-sectional study of biopsy-proven NAFLD individuals. These patients had been prospectively and consecutively enrolled from 2010 to 2015 at the Division of Gastroenterology and Hepatology of the University of Turin as part of the EU-funded FLIP/EPOS cohort. The present study includes 41 patients selected from the whole cohort (*n* = 135) according to the absence of T2DM and obesity, and with metabolomic data and fibrogenesis markers available for analysis. A flow chart of the study is provided in Supplementary Figure S1. 

Other causes of liver disease, including viral (hepatitis B and C virus infection), autoimmune, cholestatic, genetic and drug-induced diseases, were excluded, and only features of NAFLD were detected at histology. Significant alcohol consumption was excluded according to established thresholds (less than 210 g/week for males and 140 g/week for females) through direct questioning of patients and close relatives. At the time of biopsy, no clinical, biochemical or imaging-supported evidence of cirrhosis was present. A diagnosis of cirrhosis was made solely based on the histology findings. Physical examination and blood samples were collected at the time of biopsy. Obesity was defined by body mass index (BMI) equal or above 30 kg/m^2^. IR was assessed by homeostatic model assessment (HOMA)-IR according to the following formula: ((fasting plasma insulin in mU/L) × (fasting plasma glucose in mmol/L)/22.5) [13]; a HOMA-IR value higher or equal to 2.5 indicates IR. 

The study was carried out according to the principles of the Declaration of Helsinki, and it was approved by the ethics committee of the University Hospital “Città della Salute e della Scienza” of Torino (CEI/522, 23 December 2009). All patients gave signed consent for the collection of personal data in the database and for the use of blood samples for research purposes and for participation in the tracer study.

### 2.2. Analytical Determinations

Plasma samples for laboratory investigations were collected at the time of liver biopsy and stored at −80 °C for the investigations. Irisin was measured by the commercially available competitive human enzyme linked immunosorbent assay (ELISA) kit (Phoenix Pharmaceuticals, Inc., Burlingame, CA, USA) according to manufacturer’s instructions. Irisin concentration was determined with an ELISA reader at 450 nm and the final concentration was derived by the 4 parameter logistics method analysis. The intra- and inter-assay coefficients of variation were below 10% and 15%, respectively. 

The concentration of free fatty acids (FFAs), was determined by enzymatic colorimetric assays (WAKO diagnostic, Richmond, VA, USA).

Liver fibrogenesis was evaluated by interstitial matrix turnover biomarkers: N-terminal type III collagen propeptide (PRO-C3) and type VI collagen cleavage product (PRO-C6) (Nordic Bioscience competitive ELISA assays, Nordic Bioscience Laboratory, Herlev, Denmark) [14,15].

### 2.3. Histology

All liver biopsies were analyzed by a local pathologist with experience in liver disease and blinded to patients’ clinical information. The average length of liver tissue was 25 mm (range 14–45 mm) with at least 11 portal tracts. Histological features of NAFLD, including steatosis, ballooning, lobular inflammation and fibrosis, were assessed and scored according to the Clinical Research Network scoring system (NAFLD Activity Score (NAS)) [16]. The diagnosis of NASH was made according to the joint presence of steatosis, hepatocyte ballooning, and lobular inflammation. Significant fibrosis was defined as fibrosis stage equal or above 2.

### 2.4. Statistical Analysis

Data are reported as mean ± standard deviation (SD) for continuous normally distributed variables, as median and 95% confidence interval (CI) for the median for continuous not-normally distributed variables or as frequency and percentage (%) for categorical variables. Comparisons between two groups were performed by Mann–Whitney test for non-normally distributed variables and by t test for normally distributed variables. The Fisher’s exact test or the Chi-square test were used for categorical data. Spearman or Pearson correlations were performed as appropriate to evaluate the correlation between all the metabolic parameters. A multivariate regression analysis, adjusted for age and gender, was performed to assess the association between irisin levels and liver fibrogenesis. 

Values of *p* < 0.05 were considered statistically significant. All the analyses were performed with MedCalc Software bvba version 18.9.1 (Mariakerke, Belgium).

## 3. Results

A total of 41 non-diabetic, non-obese NAFLD patients were included in this study. Significant fibrosis, defined as F ≥ 2, was present in 36.6% of subjects. The clinical and biochemical characteristics of the cohort are reported in Table 1.

The median age of the whole cohort was 45 years (95% CI of the median, 41–51), 80.4% of cases male gender. Patients with F ≥ 2 had significantly higher levels of insulin and HOMA-IR. The overall prevalence of IR (by HOMA-IR ≥2.5) was higher in patients with significant fibrosis compared to those with F0/F1 fibrosis (*p* = 0.008). 

### 3.1. Histological Features Versus Circulating Irisin and Biomarkers of Fibrogenesis 

Irisin levels were higher in the population with F ≥ 2 (5.96 ng/mL (4.42–9.19) versus 2.42 ng/mL (1.73–5.95) in patients with F0/F1 fibrosis (*p* = 0.033)). Similarly, the levels of the two biomarkers of fibrogenesis, PRO-C3 and PRO-C6, were increased in NAFLD with significant fibrosis (Table 1).

Overall, NASH was found in 76% of cases, without differences between the two groups. Hepatic steatosis and ballooning degeneration were similar in the individuals with significant fibrosis vs F0/F1 fibrosis, while lobular inflammation was higher in patients with F ≥ 2 (Table 2). Circulating irisin as well as the biomarkers of fibrogenesis PRO-C3 and PRO-C6 had no correlation with hepatic steatosis, ballooning and lobular inflammation, Supplementary Table S1.

### 3.2. Circulating Irisin Versus Metabolic Profile and Biomarkers of Fibrogenesis

**Figure 1:**
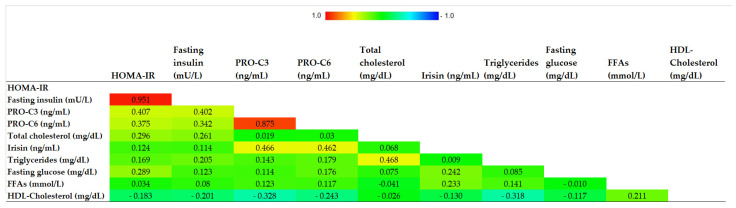
Correlogram representing the correlations between irisin levels and metabolic parameters. Abbreviations. HDL, high density lipoprotein cholesterol; FFAs, free fatty acids; HOMA-IR, homeostasis model of assessment of insulin resistance; PRO-C3, N-terminal type III collagen propeptide; PRO-C6, type VI collagen cleavage product.

Correlations between circulating irisin and metabolic parameters are reported in Figure 1.

**Figure 2:**
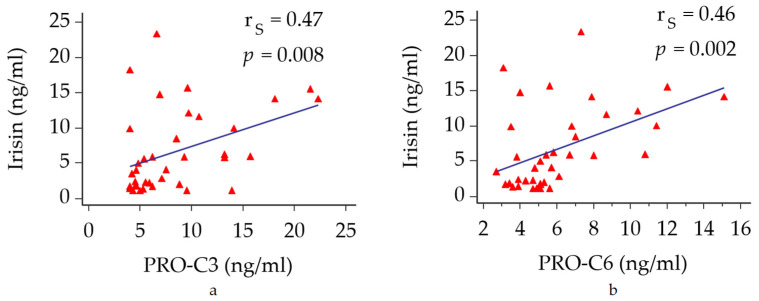
Correlations between circulating irisin with biomarkers of fibrogenesis. (**a**) correlation between irisin and PRO-C3. (**b**) correlation between irisin and PRO-C6. Abbreviations: PRO-C3, N-terminal type III collagen propeptide; PRO-C6, type VI collagen cleavage product.

No significant correlation was found between irisin levels and both glucose and lipid profile. On the other hand, circulating irisin directly correlated with both PRO-C3 and PRO-C6 levels (r_S_ = 0.47, *p* = 0.008 and r_S_ = 0.46, *p* = 0.002, respectively) (Figure 2a,b).

## 4. Discussion

We have conducted a retrospective, cross-sectional study with the aim to explore the mechanistic role of the myokine irisin in a population of biopsy-established NAFLD, in the absence of major metabolic burdens (obesity and T2DM). We found that irisin levels were significantly higher in individuals with significant fibrosis. Similarly, we found a positive correlation between circulating irisin and novel markers of collagen remodeling PRO-C3 and PRO-C6. Current evidence from the literature assesses the role of these two peptides for the quantification of liver fibrogenesis [14,15]. Our results suggest a harmonic relationship between irisin and liver fibrogenesis, which is the hepatic response to the inflammatory injury. This remarks the hypothesis that irisin is potentially a hallmark of a more severe phenotype of liver disease, which is suggested by the increased irisin levels in individuals with advanced fibrosis. 

Given the tight link between IR, liver injury and fibrogenesis, the increased irisin synthesis may represent a continuum in the damage-response role that is exerted at a mechanistic level. The increase of irisin in significant fibrosis is consistent with the results described by Petta et al. [11], where irisin was overexpressed in hepatic stellate cells of individuals with significant fibrosis. With regard to other histological features, we did not find any association between irisin and liver steatosis, differently from other studies reporting an inverse association between irisin levels and intrahepatic fat [9,10]. 

In mice, about 70% of total circulating irisin derives from SM and the remaining part mainly secreted by adipose tissue [3]. In humans, adipose tissue seems to be a relevant source of irisin synthesis only in obese individuals, due to the expansion of visceral adipose tissue [17]. In addition, a liver production of irisin has also been described [18]. In particular, in individuals with advanced liver disease, where sarcopenia is a common clinical finding, irisin levels seem to lack correlation with SM mass and with the diverse stages of liver disease severity [19]. However, similar studies remark a positive association between irisin levels and sarcopenia [20]. The heterogeneity of the study population due to either different clinical features or the severity of liver disease, in association with extraepatic co-morbidities, may significantly affect the circulating pool of irisin. In this study, it is plausible that the main source of irisin is represented by SM, potentially with a liver contribution in its synthesis, in relation to the disease activity. 

The strength of this work is the well-characterized cohort of biopsy-proven NAFLD patients who were selected for the absence of the major metabolic confounders, that could have affected the interpretations of the results. In fact, irisin seems to be involved in the regulation of IR, with conflicting results [21,22]. It seems to promote lipolysis and glucose uptake in both adipocytes and SM through GLUT4 upregulation and translocation [4]. As a result, irisin levels have been found to be associated with the risk of MetS and major cardiovascular events [23,24], but a plausible interpretation of the results is limited by the heterogeneity in MetS population, which features can significantly impact the circulating irisin pool. Male gender and age higher than 50 years are two hallmarks of higher risk for MetS development, but we did not find any difference in the distribution of irisin levels in these subgroups, when compared to the counterparts (data not shown). Our study has some limitations. The small number of patients limits the strength of the results. In addition, we did not investigate the concomitant role of SM, which is affected by IR in the setting of NAFLD. In fact, SM is a major source of peripheral IR, and the rise of sarcopenia is the hallmark of the suffering SM protein synthesis along with the metabolic disturbances. Sarcopenia may cause reduction in irisin synthesis, as described in literature, even if without strong evidence [25,26]. However, the retrospective nature of this study did not allow for further investigations at the time of liver biopsy. In conclusion, in a well-characterized cohort of non-obese, non-diabetic, biopsy-proven NAFLD individuals, increased irisin levels are found in individuals with significant fibrosis and are correlated with increased liver fibrogenesis, potentially identifying a more aggressive phenotype of liver disease. Larger studies are needed to confirm the results. The role of major metabolic confounders, such as obesity and T2DM, demands a careful selection of study populations and needs further investigations. The involvement of irisin in multiple cross talks makes it difficult to underline a target population where it would be evaluated without confounders. In addition, obesity and T2DM are the commonest features linked to NAFLD onset and progression. However, given the association with liver fibrosis in this cohort of lean individuals, is it plausible that higher levels of irisin in the absence of sarcopenia may be a potential biomarker for liver disease severity, both among regular phenotypes and for non-obese, non-diabetic patients.

## Figures and Tables

**Table 1:** Clinical, biochemical and histological characteristics of the study cohort according to degree of liver fibrosis (*n* = 41).

Variables	All (*n* = 41)	F0/F1 (*n* = 26)	F ≥ 2 (*n* = 15)	*p* Value
Age (years), median (95% CI)	45 (41–51)	44 (38–48)	51 (38–64)	0.068
BMI (kg/m^2^), median (95% CI)	25.7 (24.6–26.6)	25.6 (23.6–27.6)	25.7 (20.1–26.4)	0.705
Male/Female gender, *n* (%)	33/8 (80.4/19.6)	24/4 (85.7/14.3)	9/4 (69.2/30.8)	0.221
AST (IU/L), median (95% CI)	31 (28–36)	31 (26-35)	31 (25-58)	0.424
ALT (IU/L), median (95% CI)	48 (41–67)	47 (41–70)	53 (27–99)	0.801
Platelets (×10^9^/L), median (95% CI)	230 (216–261)	230 (206–270)	218 (201–283)	0.889
Insulin (mU/L), median (95% CI)	10.2 (9–11.8)	9.7 (8.1–10.5)	12.2 (10.1–17)	0.004
Glucose (mg/dL), median (95% CI)	94 (90–98)	92 (90–97)	97 (89–121)	0.165
HOMA-IR	2.5 (2.08–2.73)	2.15 (1.71–2.4)	2.92 (2.26–3.6)	0.012
HOMA-IR ≥ 2.5, *n* (%)	16 (39)	7 (25)	9 (69)	0.008
Total-Chol (mg/dL), median (95% CI)	184 (177–200)	184 (175–201)	190 (178–213)	0.268
HDL-Chol (mg/dL), median (95% CI)	46 (42–49)	47 (41–50)	44 (39–51)	0.492
Triglycerides (mg/dL), median (95% CI)	100 (86–117)	93 (79–118)	116 (90–178)	0.179
FFAs (mmol/L), mean (sd)	0.627 ± 0.225	0.61 ± 0.24	0.66 ± 0.2	0.449
Irisin (ng/mL), median (95% CI)	5.8 (2.87–5.96)	2.42 (1.73–5.95)	5.96 (4.42–9.19)	0.033
PRO-C3 (ng/mL), median (95% CI)	8.65 (6.32–9.64)	6.2 (4.9–8.9)	9.5 (7.7–13.6)	0.016
PRO-C6 (ng/mL), median (95% CI)	5.6 (5.1–6.74)	5.1 (4.2–5.4)	6.6 (5.6–7.9)	0.013

Note. Data are reported as mean and standard deviation, as median and 95% confidence interval of the median or as number and percentage. Abbreviations. ALT, alanine aminotransferase; AST, aspartate aminotransferase; BMI, body mass index; Chol, cholesterol; CI, confidence interval; FFAs, free fatty acids; HDL-Chol, high density lipoprotein cholesterol; HOMA-IR, homeostasis model of assessment of insulin resistance; PRO-C3, N-terminal type III collagen propeptide; PRO-C6, type VI collagen cleavage product.

**Table 2:** Histological characteristics of NAFLD patients according to liver fibrosis (*n* = 41).

Histological Features	All (*n* = 41)	F0/F1 (*n* = 26)	F ≥ 2 (*n* = 15)	*p* Value
Hepatic steatosis (%), median (95% CI)	25 (10–40)	22 (10–40)	32 (15–45)	0.583
Lobular Inflammation (0/1/2), *n* (%)	8/31/2 (19/76/5)	6/20/- (23/77/-)	2/11/2 (13/74/13)	0.005
Ballooning (0/1/2), *n* (%)	4/21/16 (10/51/39)	3/14/9 (11/54/35)	1/7/7 (6/47/47)	0.091
NASH, *n* (%)	31 (76)	19 (73)	12 (80)	0.623

Note. Data are reported as median and 95% confidence interval of the median or as number and percentage. NASH, non-alcoholic steatohepatitis.

## Supplementary Materials

The following are available online at https://www.mdpi.com/article/10.3390/jcm11030635/s1. Supplementary Figure S1: Flow chart of the study. Supplementary Table S1: Correlations between irisin, PRO-C3 and PRO-C6 with the histological features of NASH.
