# Supplementary material for: Crosstalk between Irisin Levels, Liver Fibrogenesis and Liver Damage in Non-Obese, Non-Diabetic Individuals with Non-Alcoholic Fatty Liver Disease
Source: J Clin Med. 2022 Jan 27;11(3):635. doi: 10.3390/jcm11030635 (PMC8837035; doi:10.3390/jcm11030635)
Supplement: Supplementary file 1 [file jcm-11-00635-s001.zip › jcm-1559669-supplementary.pdf]

Supplementary Figure S1. Flow chart of the study.

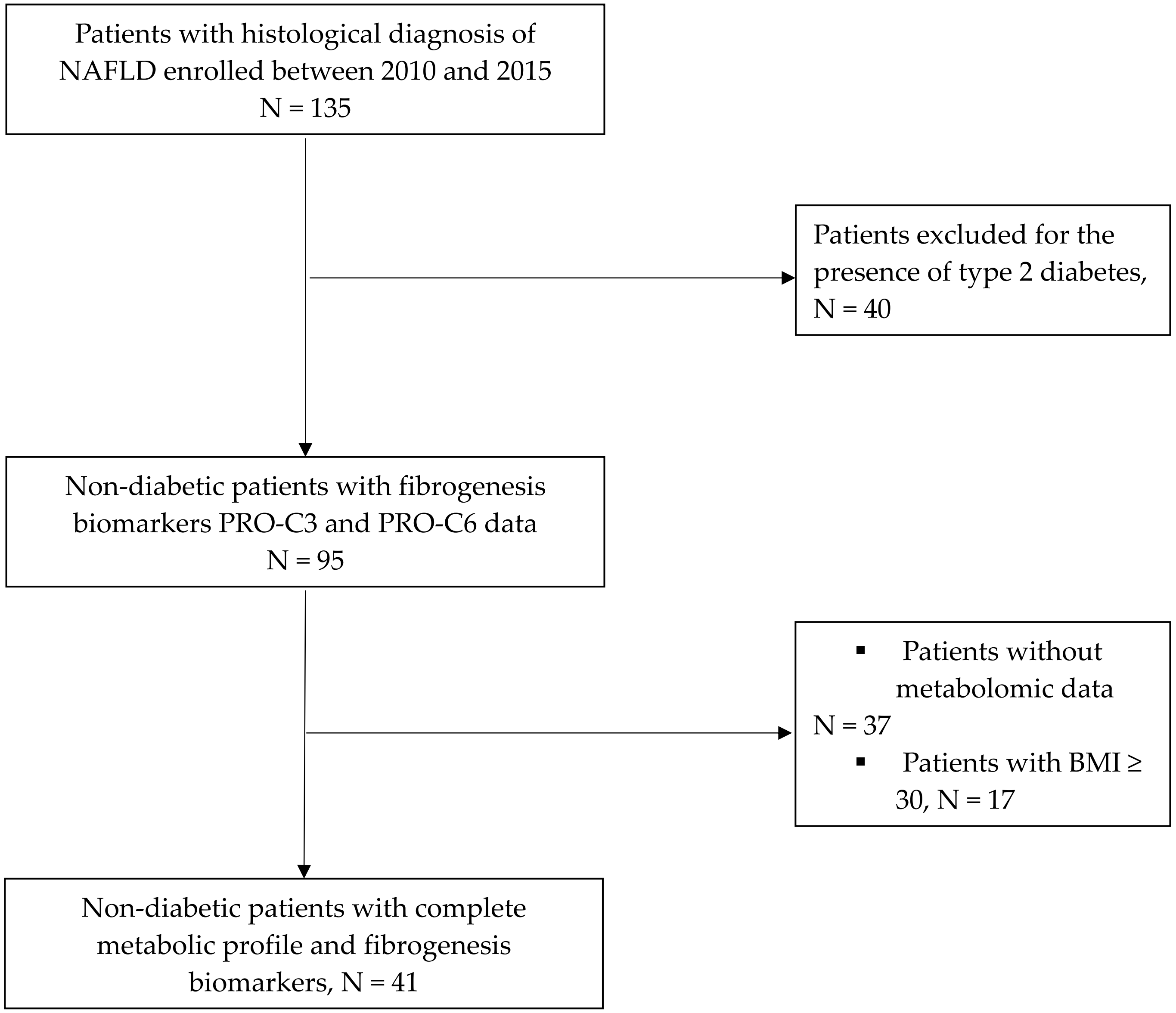

Supplementary Table S1. Correlations between irisin, PRO-C3 and PRO-C6 with the histological features of NASH.

		Irisin (ng/ml)	PRO-C3 (ng/ml)	PRO-C6 (ng/ml)	Ballooning	Lobular inflammation	Steatosis	NAS
**Irisin (ng/ml)**	rs		0.466	0.462	-0.205	-0.003	-0.169	-0.142
	P value		0.0037	0.004	0.2056	0.9844	0.296	0.3832
**PRO-C3 (ng/ml)**	rs	0.466		0.875	0.263	0.149	0.311	0.291
	P value	0.0037		<0.0001	0.1101	0.3727	0.0574	0.0765
**PRO-C6 (ng/ml)**	rs	0.462	0.875		0.113	0.132	0.305	0.211
	P value	0.004	<0.0001		0.4985	0.4289	0.0627	0.2033
**Ballooning**	rs	-0.205	0.263	0.113		0.265	0.257	0.642
	P value	0.2056	0.1101	0.4985		0.0944	0.1042	<0.0001
**Lobular inflammation**	rs	-0.003	0.149	0.132	0.265		0.226	0.762
	P value	0.9844	0.3727	0.4289	0.0944		0.1562	<0.0001
**Steatosis**	rs	-0.169	0.311	0.305	0.257	0.226		0.644
	P value	0.296	0.0574	0.0627	0.1042	0.1562		<0.0001
**NAS**	rs	-0.142	0.291	0.211	0.642	0.762	0.644	
	P value	0.3832	0.0765	0.2033	<0.0001	<0.0001	<0.0001	

Abbreviations: NAS: Non-Alcoholic Fatty Liver Disease (NAFLD) Activity Score; PRO-C3, N-terminal type III collagen propeptide; PRO-C6, propeptide of type VI collagen.
